# Functional Characterization of a Novel *TP63* Mutation in a Family With Overlapping Features of Rapp-Hodgkin/AEC/ADULT Syndromes

**DOI:** 10.1002/ajmg.a.34335

**Published:** 2011-11-08

**Authors:** Valeria Serra, Marco Castori, Mauro Paradisi, Laura Bui, Gerry Melino, Alessandro Terrinoni

**Affiliations:** 1 IDI-IRCCS Biochemistry Laboratory, c/o Department of Experimental Medicine, University of Tor VergataRome, Italy; 2 Medical Genetics, Department of Molecular Medicine, Sapienza University, San Camillo-Forlanini HospitalRome, Italy; 3 VII Pediatric Dermatology DivisionIDI-IRCCS, Rome, Italy; 4 Medical Research Council, Toxicology Unit, Hodgkin BuildingLeicester University, Leicester, UK

**Keywords:** clinical variability, frame-shift, freckling, genotype-phenotype, skin erosion

## Abstract

Heterozygous mutations in *TP63* cause a wide spectrum of autosomal dominant developmental disorders variably affecting skin, limbs, and face. *TP63* encodes p63, a protein expressed in two main isoforms (Tap63 and ΔNp63) with critical roles in both cell differentiation and development. Some analyses suggest a relationship of the mutation site to the observed clinical picture, although this link is inconsistent. This suggests an appreciable phenotypic continuity within the *TP63*-related disorders. We report a 3-month-old boy ascertained for congenital scalp erosion and mild features of ectodermal dysplasia. His mother showed full-blown characteristics of Rapp-Hodgkin syndrome plus intense abdominal and popliteal freckling. Molecular investigation identified the novel *TP63* mutation c.1697delG. We used a luciferase reporter assay to compare the effects on the p63 transactivation (TA) activity of c.1697delG with that of the p.Arg280Cys and p.Gln634X mutations, associated with ectrodactyly-ectodermal dysplasia-cleft lip/palate syndrome and isolated split hand/foot malformation, respectively. These results demonstrated complex behavior of c.1697delG in the TA of genes involved in epidermal differentiation and development and shed further light in the physiopathology of *TP63*-related disorders. © 2011 Wiley Periodicals, Inc.

## INTRODUCTION

A series of autosomal dominant developmental disorders including Rapp-Hodgkin syndrome (RHS; OMIM 129400), ankyloblepharon-ectodermal dysplasia-cleft lip/palate syndrome (AEC syndrome; OMIM 106260), ectrodactyly^1^-ectodermal dysplasia-cleft lip/palate syndrome (EEC syndrome) type 3 (OMIM 604292), acro-dermato-ungueal-lacrimal-tooth syndrome (ADULT syndrome; OMIM 103285), limb-mammary syndrome (LMS; OMIM 603543), and isolated split hand/foot malformation (SHFM) type 4 (OMIM 605289), have been associated with heterozygous mutations in the *TP63* gene [Celli et al., 1999; Ianakiev et al., 2000; Amiel et al., 2001; McGrath et al., 2001; van Bokhoven et al., 2001; Kantaputra et al., 2003]. These discoveries argued for molecular lumping in contrast to the clinical splitting that characterized the history of these conditions. Currently, allelic heterogeneity for the same condition and marked clinical variability for single mutations are the rule within the wide spectrum of TP63-associated disorders [Rinne et al., 2007].

TP63 encodes for p63, which is a key molecule in both development and carcinogenesis [Melino et al., 2003]. Its structure comprises five domains, including the transactivation (TA) domain, DNA-binding domain (DBD), oligomerization domain [Levrero et al., 1999], sterile-α-motif (SAM) domain and the transactivation inhibitory (TI) domain [Ghioni et al., 2002]. p63 expression is driven by two promoters giving rise to two isoforms containing (TAp63) or lacking (ΔNp63) the TA domain [Yang et al., 1998]. Both isoforms are, in turn, differentially spliced at the 3′ end with the formation of three proteins with distinct C-termini (α-, β-, and γ-isoforms) with only the α-isoform containing the SAM and TI domains [Celli et al., 1999]. The TI domain interacts with the TA domain and masks residues relevant for TA, thus suppressing TAp63-mediated TA [Yang et al., 1998; Serber et al., 2002]. Some genotype–phenotype correlations, such as EEC syndrome associating with mutations in the DBD, and RHS and AEC syndromes with mutations preferentially clustering in the SAM and TI domains [Rinne et al., 2009; Clements et al., 2010], suggest a complex and heterogenous pathogenesis for the *TP63*-related disorders.

We report on a 3-month-old boy and his affected mother displaying a highly variable phenotype and sharing a previously unreported *TP63* mutation. Functional studies of the identified mutation shed some additional light on the molecular physiopathology of *TP63*-related disorders.

## CLINICAL AND MOLECULAR STUDY

This family came to our attention for the presence of congenital and persistent scalp dermatitis in a 3-month-old boy born to 34-year-old mother and her 38-year-old healthy and unrelated husband. The proband was born at term from uneventful pregnancy and spontaneous delivery. Birth weight was 3,300 g (∼50th centile), length 51 cm (∼50th centile) and OFC 34.5 cm (25th–50th centile), and Apgar score 8^1^/10^5^. At birth, an extensive skin erosion was noted that involved the vertex, the parietal, and part of the occipital regions. This lesion persisted in the following weeks without spontaneous regression. His eyelids were reportedly normal at birth. The patient held his head up at 1.5 months and was regularly breastfed.

**FIG. 1:**
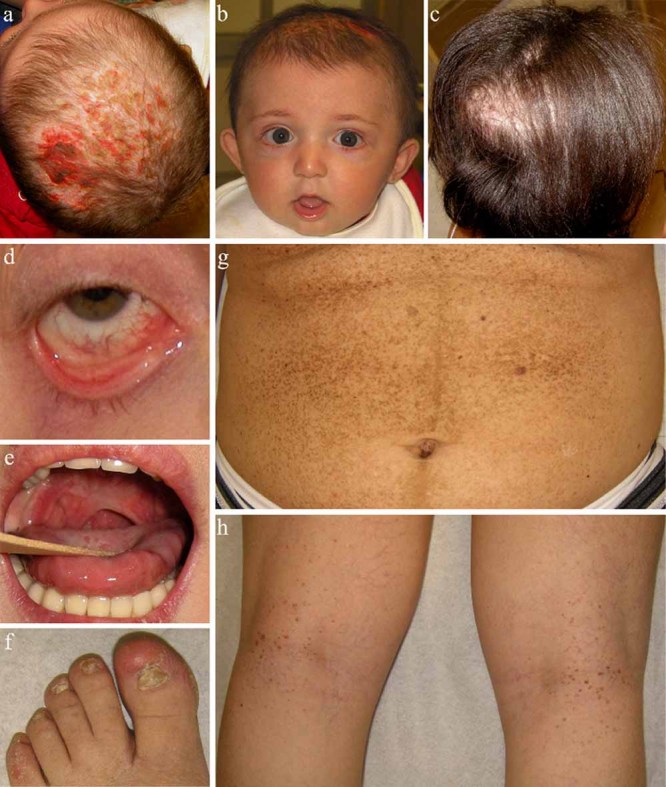
Clinical features of the family. The child had extensive skin erosion of the interparietal region (**a**) and mild facial dysmorphisms (**b**), including downslanted palpebral fissures, sparse eyelashes and eyebrows, short nose, narrow mouth, and protruding ears compatible with the diagnosis of ectodermal dysplasia. His mother had non-cicatricial alopecia and hypotrichosis (**c**), a small right lachrymal puncta (**d**), repaired cleft palate (**e**), toenail dystrophy (**f**), and abdominal (**g**) and popliteal (**h**) freckling.

At examination, at 3 months, a large cutaneous lesion extending over the interparietal region, and scattered by crusts and areas of cicatricial alopecia were evident (Fig. 1a). The skin of the remaining body surface appeared somewhat thinner than normal, diffusely xerotic, and marked by a fine and persistent erythematous reticulum resembling *cutis marmorata*. Mild dystrophic changes were also evident on nails of fingers and toes. The face appeared slightly dysmorphic with downslanted palpebral fissures without synechiae, sparse eyelashes and eyebrows, short nose, narrow mouth, and protruding ears (Fig. 1b). Oral cavity examination was unremarkable. Neurological status was normal.

The mother also showed typical features of hypohydrotic ectodermal dysplasia. At birth, she displayed cleft palate with a well-formed upper lip. Diffuse xerosis and erythrodermic changes were additional remarkable features noted at birth. No congenital skin erosion was reported. The patient complained of generalized hypohydrosis and tendency to sunburns, but she tolerated heat well. Scalp hair, body hair, and nails grew slowly, and were thin and overtly dystrophic. Since childhood, the patient showed diffuse freckling of the back and abdomen. Many teeth of the primary and secondary dentitions were missing and most of those erupted were small and “peg-shaped”. For this she requested fixed prosthesization at 25 years of age.

On physical examination, her skin was thin and dry with periorbital darkening. Scalp hair, pubic hair, eyelashes, and eyebrows were sparse. She had no signs of scalp inflammation (Fig. 1c). No axillary hair was present. The lachrymal punctae were small (Fig. 1d). Despite diffuse conjunctival injection, the patient did not complain of epiphora, recurrent conjunctivitis, or photophobia. Oral examination showed absent uvula and the remnants of the surgical repair of cleft palate (Fig. 1e). The toenails (Fig. 1f) and fingernails were dystrophic and brittle. Diffuse freckling was present on her back and abdomen, especially above the umbilicus (Fig. 1g). Freckling of the popliteal region was also noted (Fig. 1h). Cognitive development appeared unremarkable. The patient did not complain any problem related to menses or swallowing. A recent hearing evaluation was normal. No other family members were available for study.

**FIG. 2:**
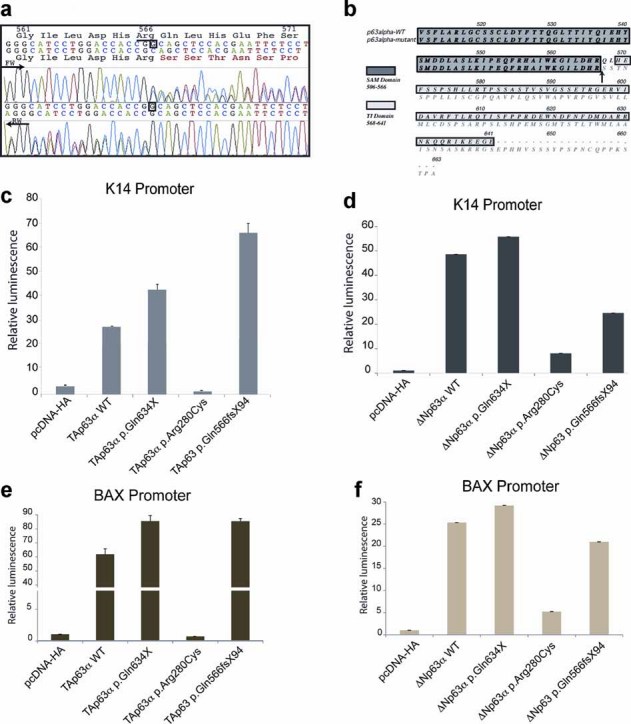
Analysis of *TP63* gene. *TP63* sequence chromatogram of the child showing the heterozygous c.1697delG mutation (**a**). Comparison of the predicted mutant C-terminal sequence and the wild type p63 protein (**b**). Luciferase reporter assay comparing the *K14* and *BAX* transcriptional activity modulated by both p63 isoforms (i.e., TAp63α and ΔNp63α), and carrying the wild-type sequence (WT) or specific mutations, including the previously reported p.Gln634X and p.Arg280Cys mutations, and the novel mutation, which predicts p.Gln566fsX94 (**c**–**f**). *K14* promoter activity related to TAp63α (c) and ΔNp63α (d) isoforms. *BAX* promoter activity related to TAp63α (e) and ΔNp63α (f) isoforms. pcDNA-HA stands for empty expressing vector used as a control. Data are presented as fold change over control. Three independent experiments were performed (mean + /−SD, n = 3).

After obtaining informed consent, the DNA of both patients was extracted from peripheral blood lymphocytes and used for PCR amplification and direct sequencing of known *TP63* exons. A heterozygous c.1697delG variant in exon 14 was identified in the child (Fig. 2a) and his mother. Mutations were numbered according to reference sequence NM_003722.4.

## FUNCTIONAL STUDY

The c.1697delG mutation was predicted to cause p.Gln566fsX94. The new terminal sequence lacked homology with the TI domain (spanning from amino acids 568 to 641 in the wild type sequence), as well as with any known proteins, according to the GenBank, PDB, SwissProt, PIR and PRF databases (Fig. 2b).

To investigate the functional effects of the mutation, wild-type TAp63α and ΔNp63α vectors generated in Saos 2 cell lines [Zocchi et al., 2008] were mutagenized with the insertion of the mutation described here in their coding region by using the following oligos: forward 5′-CTGGACCACCGCAGCTCCACG-3′ and reverse 5′-CGTGGAGCTGCGGTGGTCCAG-3′. In previous studies, these vectors proved useful for analyzing the transcriptional activity of mutant p63 proteins on known promoters. For the present study, a luciferase reporter assay was performed in HEK 293 cells as described [Candi et al., 2006a] using the firefly luciferase gene under the control of the *K14* promoter, which modulates the expression of a protein physiologically regulated during keratinocyte differentiation (Fig. 2c,d), and the *BAX* promoter, which influences the expression of a gene important in cell cycle and apoptosis especially during embryogenesis (Fig. 2e,f) [Candi et al., 2006b]. The effects of the mutation identified here were compared with the wild-type sequence as well as with two known mutations, including p.Arg280Cys, classically associated with EEC syndrome, and p.Gln634X, which causes SHFM.

Similar to previous results [Brown et al., 2011], the study of the *K14* promoter demonstrated an increase of the transcriptional activity (i.e., gain of function) for the predicted TAp63α p.Gln566fsX94 construct. This effect was higher than the TAp63α p.Gln634X mutant, located in the same p63 domain (Fig. 2c). Conversely, the luciferase reporter assay analysis for the ΔNp63α isoform indicated significantly lower TA activity (i.e., loss of function) for the predicted p.Gln566fsX94 mutant with respect to the wild-type isoform (Fig. 2d). As expected, the p.Arg280Cys mutant showed no activity in both TAp63α and ΔNp63α isoforms (i.e., loss of function; Fig. 2c,d).

Experiments performed on the *BAX* promoter showed gain of function for the predicted TAp63α p.Gln566fsX94 mutant, similar to that observed for the p.Gln634X mutation (Fig. 2e). Instead, the assay performed with the predicted ΔNp63α p.Gln566fsX94 mutant showed TA activity comparable to the wild-type allele (Fig. 2f). In both cases, the p.Arg280Cys mutation displayed a strong decrease in TA activity (Fig. 2e,f).

## DISCUSSION

The combination of hypotrichosis, nail dystrophy, hypohydrosis, hypoplastic lachrymal punctae, and cleft palate in the mother of the proband is characteristic of RHS. However, diffuse freckling, observed on her trunk and popliteal regions, is atypical in RHS, while it is a cardinal feature of ADULT syndrome [Propping and Zerres, 1993]. A further element of variability is the extensive congenital scalp dermatitis with underlying skin erosion in the child reported here. In fact, such a presentation has been only occasionally reported in RHS, while it is more typical of AEC syndrome [Clements et al., 2010]. Notably, in the child, the scalp defect represented the primary reason of referral. This family illustrates the poor specificity of most features observed within the *TP63*-related disorders phenotypic spectrum and the utility of following the manifestations over time. We speculate that there will be an increasing number of reports describing intermediate phenotypes in the *TP63*-related disorders.

The identified mutation is located in the TI domain. This domain inhibits the TA domain located in the N-terminus. Mutations disrupting the TI domain usually result in perturbation of the intra-molecular binding between the TI and TA domains with consequent loss of inhibition of the TA activity of p63 [Serber et al., 2002]. Therefore, the presumed consequence of TI mutations is gain of function. However, the existence of two isoforms (i.e., TAp63 and ΔNp63) complicates this model. Accordingly, in the present family, functional studies indicated that the mutated TAp63α isoform results in gain of function activity on both *K14* and *BAX* promoters, which are involved in distinct cellular pathways (i.e., cell cycle and differentiation, respectively). These results are consistent with other mutations located in the same domain (i.e., p.Gln634X) and responsible of SHFM [Brown et al., 2011]. The c.1697delG mutation is consistent with the previously reported c.1787delG and c.1859delA mutations located in the same domain and variably associated with both AEC syndrome and RHS [Clements et al., 2010]. This finding supports the clinical and molecular continuum of these two disorders, which may better be considered the result of phenotypic variability of a single condition.

However, discordant results were obtained for the ΔNp63α isoform. In particular, values comparable with the wild-type allele were obtained for the *K14* promoter, while a slight, though significant loss of function was appreciated for the *BAX* promoter. This is in contrast with the findings for p.Gln634X, which demonstrates a gain of function for the *BAX* promoter in the ΔNp63α isoform. These results are consistent with the p.Arg280Cys mutation, which causes loss of function. This discrepancy may partly explain the dichotomy of the phenotype observed in this family and that associated with p.Gln634X. In fact, p.Gln634X specifically affects limb development and results in isolated SHFM, while the predicted p.Gln566fsX94 mutation causes ectodermal dysplasia with incompletely penetrant cleft palate and normal appendicular structures. One limitation of the present study is that the mutant allele could be degraded due to nonsense-mediated mRNA decay, which would predict that the allele is a pure null. However, this hypothesis was not tested in this work.

Experiments investigating the stability of p63 mutants showed that mutations of the DNA-binding and SAM domains and causing ADULT syndrome, EEC syndrome and LMS, usually show absent or drastically reduced transcriptional activity, as recapitulated by the results for p.Arg280Cys. This phenomenon is not due to altered localization, since all investigated mutants exhibited normal nuclear staining [Brown et al., 2011]. Conversely, some C-terminal mutants such as p.Gln634X, retain transcriptional competence. This could explain the lack of a skin phenotype. The pathogenic consequences of these mutants could be the over-expression of target, dosage-sensible genes involved in limb development. That the predicted p.Gln566fsX94 mutation is associated with a skin phenotype may mirror the complementary effects of the mutant ΔNp63α isoform on the transcription of developmental genes, such as *BAX*. The transcriptional activity profile of the predicted p.Gln566fsX94 mutation, in contrast to that for p.Gln634X, may be shared with other C-terminus mutations causing RHS and AEC syndrome, which lack limb malformations [Clements et al., 2010]. Further studies are needed to explain the complex post-transcriptional network influencing the phenotypic consequences of *TP63* mutations.

## Footnotes

1 The term “ectrodactyly” has been replaced by the term “split hand” in the current dysmorphology terminology [Biesecker et al., 2009]
